# Concordance between environmental resistomes and pathogenic phenotypes: a case study of multidrug-resistant *Klebsiella pneumoniae* in a drinking water source in Guizhou, China

**DOI:** 10.3389/fmicb.2026.1810806

**Published:** 2026-05-07

**Authors:** Wanning Qian, Ao Han, Abdullah M. S. Al Hatmi, Yanyan Wang, Muhammad Rafiq, Guzhen Cui, Shaoqin Zhou, Shijun Li, Yingqian Kang

**Affiliations:** 1 Key Laboratory of Environmental Pollution Monitoring and Disease Control, School of Public Health, Ministry of Education, Guizhou Medical University, Guiyang, China; 2 Guizhou Key Laboratory of Microbiome and Infectious Disease Prevention, Institution of One Health Research, Guizhou Medical University, Guiyang, China; 3 Natural & Medical Sciences Research Center, University of Nizwa, Nizwa, Oman; 4 Department of Microbiology, Balochistan University of IT, Engineering and Management Sciences (BUITEMS), Quetta, Pakistan; 5 Guizhou Center for Disease Control and Prevention, Guiyang, Guizhou, China

**Keywords:** antibiotic resistance, antibiotic resistance genes, drinking water sources, *Klebsiella pneumoniae*, metagenomics

## Abstract

**Introduction:**

The persistence of antibiotic resistance in aquatic environments poses a public health concern, particularly when drinking water sources act as reservoirs for multidrug-resistant opportunistic pathogens. However, the linkage between environmental resistomes and the resistance phenotypes of cultivable bacteria remains under-characterized. This case study investigated this relationship in a karst drinking water source in Guizhou, China.

**Methods:**

Surface water samples from seven sites were analyzed for antibiotic residues using LC-MS/MS. Metagenomic sequencing was conducted on selected contamination hotspots to characterize microbial communities and antibiotic resistance genes (ARGs). Cultivable bacteria were isolated, identified via 16S rRNA sequencing, and tested for antimicrobial susceptibility. To validate resistance mechanisms, a multidrug-resistant *Klebsiella pneumoniae* isolate was analyzed for *tetA* expression using RT-qPCR.

**Results:**

Antibiotic residues were detected across all sites, with sulfonamides and tetracyclines being the most prevalent. Consistent with this chemical pressure, metagenomic analysis identified corresponding ARGs, including *sul1* and *tet(Q)*, which functionally clustered with mobile genetic elements. From the contaminated matrix (sample W2), a multidrug-resistant *Klebsiella pneumoniae* strain (B8) was recovered. Mechanistic validation revealed a 2.78-fold upregulation of the *tetA* efflux pump gene in this strain.

**Discussion:**

These findings demonstrate a concordance among chemical selection pressures, environmental resistomes, and active resistance phenotypes. The results indicate that drinking water sources can harbor and maintain clinically relevant resistant bacteria, supporting the implementation of integrated surveillance strategies to evaluate biological risks.

## 1 Introduction

The widespread use of antibiotics in clinical, agricultural, and veterinary settings has led to their continuous release into the environment, creating a persistent ecological challenge (Adeoye and Keenum, 2025). Aquatic ecosystems, in particular, serve as major sinks for these contaminants. Previous studies have shown that antibiotics enter surface water and groundwater through multiple pathways (Liu et al., 2025), including agricultural runoff, aquaculture discharge, and effluents from wastewater treatment plants, largely because of incomplete metabolism and the limited removal efficiency of conventional treatment processes (Otaibi et al., 2024). Accordingly, even at trace concentrations in the ng/L to μg/L range, antibiotic residues can exert sustained selective pressure on indigenous microbial communities. This chronic exposure promotes the maintenance and dissemination of antibiotic resistance genes (ARGs), often facilitated by mobile genetic elements (MGEs) such as transposons and plasmids (Abimbola et al., 2023).

Although drinking water treatment processes, including coagulation, sedimentation, filtration, and disinfection, are designed to ensure microbiological safety, accumulating evidence indicates that they are not (Thabang et al., 2023). Antibiotic residues and extracellular ARGs can persist in source water, potentially bypassing treatment barriers or accumulating in distribution systems. These resistance-related factors are dynamic components of a coupled ecological cycle involving water, soil, and human or animal hosts (Yingjie et al., 2022). For instance, the use of contaminated source water for irrigation can enrich the resistome of soil and rhizosphere microbiota, subsequently entering the food web. Therefore, assessing the risk of drinking water sources requires a perspective that transcends simple compliance with water quality standards, focusing instead on the ecosystem-level dynamics of the resistome (Alison et al., 2016).

A significant limitation in current environmental microbiology is the methodological fragmentation of risk assessment. Many studies focus on single-dimensional analyses: solely monitoring chemical residues, exclusively profiling metagenomic DNA, or isolating bacteria without genomic context (Liu et al., 2021). While metagenomics offers a comprehensive inventory of the “potential” resistome, it often fails to confirm whether these genes are present in viable, clinically relevant pathogens. Conversely, culture-based methods isolate specific strains but may miss the broader genetic reservoir of the community. There is a paucity of research that seamlessly integrates chemical contamination profiles with environmental resistome structures and subsequently validates these findings through the phenotypic and molecular mechanisms of cultivable strains (Shi et al., 2009). This gap is particularly important in drinking water sources, where opportunistic pathogens such as *Klebsiella pneumoniae* pose a direct public health threat (Si et al., 2020).

To address this disconnect, this research was designed as an integrated case study in a specific region of Guizhou Province, China. Rather than relying on broad inferences, we aimed to provide a multi-level evidence chain linking environmental pressures to bacterial physiology (Annaleise et al., 2021). By combining liquid chromatography-tandem mass spectrometry (LC-MS/MS) quantification of antibiotic residues, metagenomic profiling of the microbial community, and targeted isolation of key pathogens, we sought to determine whether the environmental resistome is reflected in active resistance in cultivable bacteria (Chang et al., 2023). Specifically, we isolated a multidrug-resistant *Klebsiella pneumoniae* strain and experimentally validated *tetA* expression to assess concordance between the environmental gene reservoir and pathogen adaptive mechanisms.

## 2 Materials and methods

### 2.1 Study area and sample collection

To investigate antibiotic resistance in a karst hydrologic system, surface water samples were collected from seven designated monitoring sites (W1–W7) representing typical municipal drinking water sources in Guizhou Province, China. To comply with local public health infrastructure confidentiality protocols, the exact geographical coordinates and reservoir names have been anonymized. Sampling was conducted longitudinally from October 2024 to July 2025 to capture seasonal variations. At each site, water samples were collected in triplicate using pre-sterilized 500 mL polyethylene bottles. Immediately after collection, the samples were packed with ice packs inside insulated cooler boxes to strictly maintain a continuous transport temperature of 4 °C, thereby minimizing biological activity during transit to the laboratory. Upon arrival, each sample was divided into two portions: one was processed immediately for the isolation of cultivable bacteria, while the other was filtered through 0.45 μm membrane filters and stored at −20 °C for the subsequent analysis of antibiotic residues (Min et al., 2020).

### 2.2 Determination of antibiotic concentrations through LC–MS/MS

Antibiotic residues from five major therapeutic classes (β-lactams, macrolides, quinolones, sulfonamides, and tetracyclines) were quantified using an Agilent 6,460 triple quadrupole LC-MS/MS system. Prior to extraction, frozen samples were gradually thawed at 4 °C. A 500 mL aliquot of each sample was filtered through a 0.45 μm membrane and enriched using Oasis HLB solid-phase extraction (SPE) cartridges (Xiang et al., 2020). The SPE cartridges were pre-conditioned sequentially with methanol and ultrapure water. Following sample loading, the target analytes were eluted and evaporated to near-dryness under a gentle nitrogen stream. The residues were subsequently reconstituted in a methanol-water mixture (1:1, v/v), passed through a 0.22 μm syringe filter, and transferred to vials for instrumental analysis.

Chromatographic separation was performed on a C18 reversed-phase column maintained at 30 °C. The binary mobile phase consisted of 0.1% formic acid in water (Phase A) and 0.1% formic acid in methanol (Phase B), delivered at a flow rate of 0.3 mL/min under a gradient elution profile. The sample injection volume was 5 μL. Mass spectrometry was operated using an electrospray ionization (ESI) source in multiple reaction monitoring (MRM) mode, with switching between positive and negative polarities (Andrew and Christina, 2020). Analytes were identified and quantified based on specific precursor-to-product ion transitions using the external standard method. Calibration curves exhibited high linearity (*R*^2^ > 0.99) over a concentration range of 0.5 to 200 μg/L. Method validation confirmed that the recovery, precision, and detection limits met the standard analytical requirements for trace environmental analysis.

### 2.3 Isolation and purification of cultivable bacteria

A culture-dependent approach utilizing serial dilution and spread-plating was employed to recover bacterial isolates from the source water. Samples were processed immediately upon laboratory receipt to maintain cell viability. A tenfold serial dilution (10^−1^ to 10^−4^) was prepared using sterile phosphate-buffered saline (PBS).To maximize the recovery of both copiotrophic and oligotrophic bacteria, 200 μL aliquots of each dilution were evenly spread in triplicate onto Luria-Bertani (LB) and R2A agar plates. These plates were incubated inverted at 28 °C for 36–48 h. Morphologically distinct colonies were selected and purified through three successive rounds of streaking on fresh LB agar to obtain stable, homogeneous isolates. Finally, the purified strains were assigned identification codes, cryopreserved in glycerol stocks, and inoculated into liquid LB broth for downstream identification and phenotypic characterization (Lyu et al., 2020).

### 2.4 Molecular identification via 16S rRNA sequencing

The taxonomic identity of the purified isolates was determined via 16S rRNA gene sequencing. Single colonies exhibiting robust growth were transferred into 5 mL of LB broth and incubated at 37 °C with agitation for 6 h. Genomic DNA was extracted using a commercial bacterial DNA extraction kit (Tiangen Biotech, China) to serve as the template for polymerase chain reaction (PCR). The 16S rRNA gene was amplified using the universal primer pair 27F (5′-AGAGTTTGATCCTGGCTCAG-3′) and 1492R (5′-GGTTACCTTGTTACGACTT-3′). The thermal cycling protocol consisted of an initial denaturation at 95 °C for 5 min, followed by 30 cycles of amplification (95 °C for 30 s, 55 °C for 30 s, and 72 °C for 90 s), and a final extension at 72 °C for 7 min. PCR products were verified via 1% agarose gel electrophoresis prior to being sequenced by General Biol (Anhui, China). The isolates were taxonomically identified by comparing the obtained sequences against the National Center for Biotechnology Information (NCBI) database using the Basic Local Alignment Search Tool (BLAST).

### 2.5 Phenotypic antimicrobial susceptibility testing

Antimicrobial susceptibility profiles of the bacterial isolates were evaluated using the disk diffusion assay, adhering to the protocols outlined by the Clinical and Laboratory Standards Institute (CLSI) M100-Ed33 guidelines. Purified colonies were suspended in 5 mL of LB broth and incubated at 37 °C with agitation for 6–8 h. The bacterial suspension density was adjusted to the 0.5 McFarland standard, corresponding to an optical density at 600 nm (OD600) of 0.08–0.10. A sterile cotton swab was used to spread a confluent bacterial lawn onto Mueller-Hinton (MH) agar plates. After the agar surface dried, ten specific antibiotic-impregnated disks were applied: penicillin (10 U), ampicillin (10 μg), ceftriaxone (30 μg), tetracycline (30 μg), erythromycin (15 μg), ciprofloxacin (5 μg), trimethoprim-sulfamethoxazole (1.25/23.75 μg), chloramphenicol (30 μg), lincomycin (10 μg), and gentamicin (120 μg). The plates were statically incubated at 37 °C for 18–24 h. The diameters of the inhibition zones were then measured, and the strains were categorized as susceptible, intermediate, or resistant based on CLSI breakpoints. Isolates exhibiting multidrug resistance (MDR), particularly those resistant to tetracycline, were selected for downstream molecular analysis to verify the expression of specific resistance genes.

### 2.6 Quantification of resistance gene expression via RT-qPCR

Among the isolated bacteria, *Klebsiella pneumoniae* strain B8 was selected for molecular validation due to its clinical significance and distinct MDR phenotype, particularly against tetracycline. To elucidate the molecular basis of this resistance, the expression of the *tetA* gene was quantified. This gene encodes a primary tetracycline efflux pump in Gram-negative bacteria and is commonly associated with plasmid-mediated horizontal transfer in aquatic systems (Marathe et al., 2018). Total RNA was extracted from *Klebsiella pneumoniae* strain B8 and reverse-transcribed into complementary DNA (cDNA). Gene expression profiling was performed using SYBR Green-based reverse transcription quantitative PCR (RT-qPCR), with the 16S rRNA gene serving as the endogenous housekeeping control to normalize transcriptional variability. Relative expression changes were calculated using the 2^−ΔΔ*Ct*^ method. The specific primers used were *tetA-F* (5′-GCTACATCCTGCTTGCCTTC-3′) and *tetA-R* (5′-CATAGATCGCCGTGAAGAGG-3′), yielding a 110 bp amplicon. Each 20 μL reaction mixture contained 10 μL of SYBR Green Premix, 2 μL of cDNA template, 0.8 μL of each primer, and nuclease-free water. The thermal cycling conditions included an initial denaturation at 95 °C for 30 s, followed by 40 cycles of 95 °C for 5 s and 60 °C for 30 s. All samples were analyzed in triplicate across three independent experiments. Plasmids harboring the *tetA* gene and no-template controls (NTC) were included as positive and negative controls, respectively. Amplification efficiencies ranged from 90% to 105%, with standard curve R^2^ values exceeding 0.99.

### 2.7 Metagenomic sequencing and bioinformatic analysis

#### 2.7.1 DNA extraction, library construction, and sequencing

Based on preliminary LC-MS/MS profiling, samples W1 and W2 were prioritized for deep shotgun sequencing due to their complex multi-class antibiotic contamination signatures (predominantly sulfonamides and tetracyclines), representing critical environmental pressure scenarios. Total genomic DNA was extracted using the FastDNA Spin Kit for Soil (MP Biomedicals, USA) and quantified with a Qubit 4.0 fluorometer. Approximately 200 ng of DNA was randomly fragmented to 300–350 bp using a Covaris ultrasonicator. Sequencing libraries were constructed via standard end repair, phosphorylation, A-tailing, adapter ligation, and PCR amplification. Following quality validation with an Agilent 2100 Bioanalyzer, the libraries were sequenced on an Illumina NovaSeq platform (PE150 mode) (Grenni et al., 2018).

#### 2.7.2 Quality control and assembly

Raw sequencing data were processed using bcl2fastq (v2.17.1.14). Low-quality reads and adapters were removed using Cutadapt (v1.9.1) by trimming bases with quality scores below 20 and excluding reads shorter than 75 bp or containing >10% ambiguous bases. After filtering host contamination with BWA (v0.7.12), the high-quality clean reads were assembled *de novo* using MEGAHIT (v1.1.3) via a multi-k-mer strategy (*k*-mers: 59, 79, 99, 119, 141).

#### 2.7.3 ORF prediction and construction of a non-redundant gene set

Open reading frames (ORFs) were predicted using Prodigal (v3.02). To construct a non-redundant gene catalog (Unigenes), predicted sequences from all samples were clustered using MMseqs2 with thresholds of ≥95% sequence identity and ≥90% coverage. Clean reads were then mapped back to the Unigenes using SoapAligner (v2.21) to calculate relative gene abundances (Wang et al., 2017).

#### 2.7.4 Taxonomic annotation

Taxonomic classification was assigned by aligning the Unigene protein sequences against the NCBI non-redundant (NR) protein database using Diamond (v0.8.15.77) with an e-value threshold of < 1e−5.

#### 2.7.5 Functional and resistome annotation

Functional annotation was conducted by aligning Unigene sequences against the Kyoto Encyclopedia of Genes and Genomes (KEGG), evolutionary genealogy of genes: Non-supervised Orthologous Groups (eggNOG v4.5), and Carbohydrate-Active enZYmes (CAZy) databases using BLASTP (v2.2.31+) with an e-value threshold of < 1e−5. Furthermore, ARGs and virulence factors were identified by aligning sequences against the Comprehensive Antibiotic Resistance Database (CARD) and the Virulence Factor Database (VFDB), respectively, employing a rigorous threshold of ≥90% sequence identity and an e-value < 1e−5.

#### 2.7.6 Statistical analysis

Statistical analyses were performed using SPSS Statistics 26.0 (IBM, USA). For the RT-qPCR assay, differences in relative gene expression levels between the experimental isolate and the reference control were evaluated using independent samples *t*-tests, with statistical significance defined at *p* < 0.05. Ecological data visualization and diversity analyses were conducted in R (v4.1.2). Where necessary for visual scaling in heatmaps, raw data for antibiotic concentrations were log-transformed. The *vegan* package was used to compute alpha (Chao1, Shannon) and beta diversity (Bray-Curtis dissimilarity) metrics, while principal coordinates analysis (PCoA) plots and clustered heatmaps were generated using the *ggplot2* and *pheatmap* packages, respectively.

## 3 Results

### 3.1 Occurrence of antibiotics in drinking water samples

**Figure 1:**
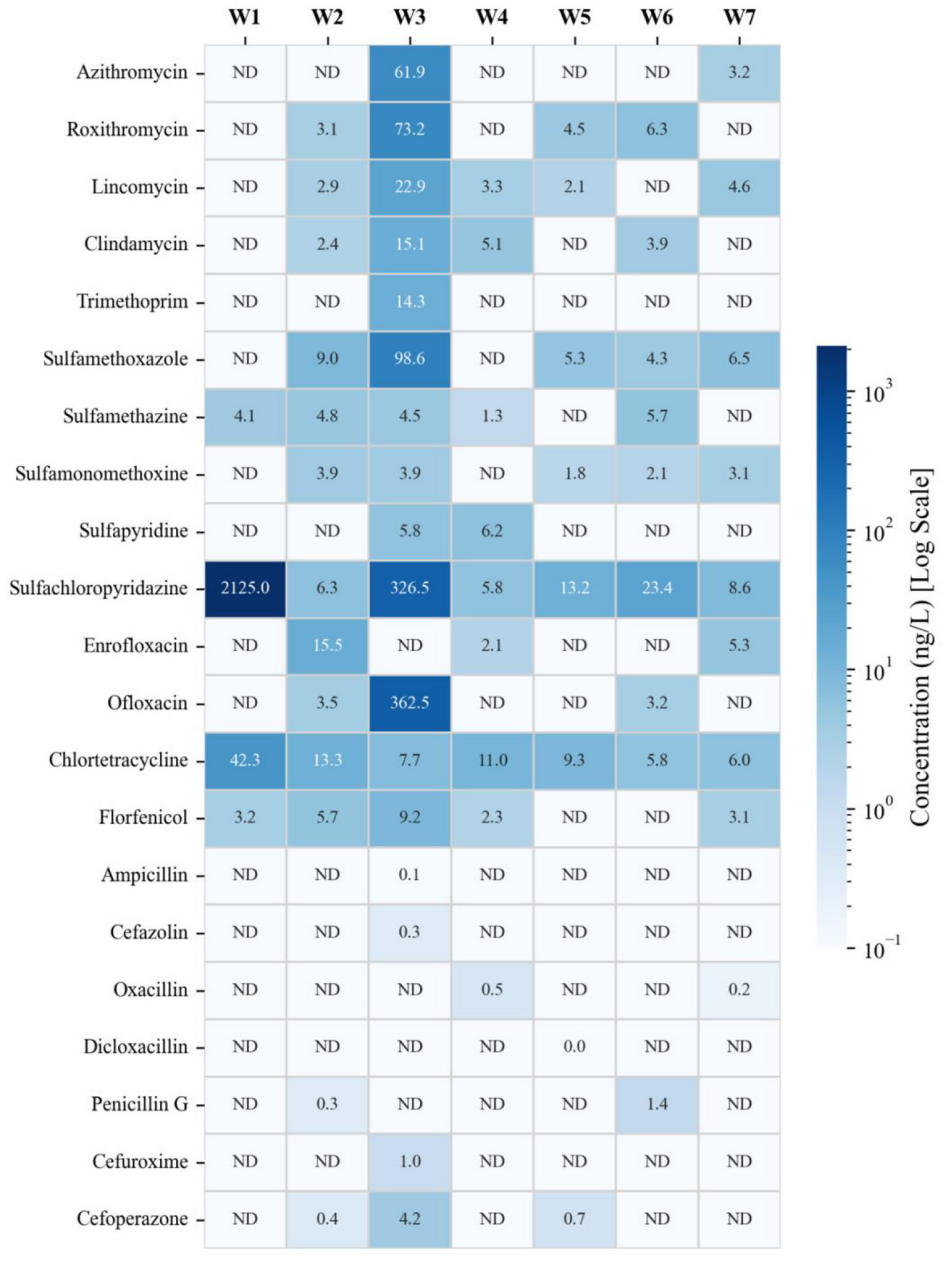
Heatmap distribution of antibiotic residues detected across seven drinking water source sampling sites (W1–W7). The concentrations (ng/L) of detected antibiotics are visualized using a logarithmic color scale (Blues). Darker blue indicates higher concentrations, while lighter shades represent lower levels. “ND” denotes not detected (concentration below the limit of quantification). The clustering of high concentrations in samples W1 and W2 highlights the spatial heterogeneity of contamination, identifying them as hotspots for resistome investigation.

A comprehensive LC-MS/MS screening was conducted across seven designated monitoring points (W1–W7) to characterize the specific chemical pressures within the studied karst hydrologic system. As visualized in the concentration heatmap (Figure 1), the analysis confirmed the ubiquity of antibiotic residues, identifying 24 distinct compounds spanning five major therapeutic classes: macrolides, sulfonamides, tetracyclines, quinolones, and β-lactams.

Significant differences were observed in both the types and concentrations of antibiotics detected among the sampling sites. The heatmap distinctly highlights the spatial heterogeneity of the contamination, identifying Samples W1 and W2 as prominent hotspots with the most complex multi-class antibiotic signatures, justifying their selection for subsequent metagenomic sequencing. Specifically, Sample W1 was characterized by a high abundance of sulfonamide antibiotics, with sulfachloropyridazine reaching the highest recorded quantity in the study (2,125.0 ng/L), alongside high quantities of chlortetracycline (42.3 ng/L). Sample W2 demonstrated a more varied multi-class coexistence profile. High amounts of specific antibiotics, such as ofloxacin (362.5 ng/L) and various macrolides, were also recorded in Sample W3, further emphasizing the spatial variation across locations. Samples W4–W7 generally displayed lower background concentrations and fewer types of antibiotics.

Overall, tetracyclines and sulfonamides were the most frequently detected classes across all samples, which aligns with their extensive veterinary and agricultural usage in China and their high propensity to enter and persist in surface waters via environmental runoffs (Guo et al., 2022). Conversely, β-lactam antibiotics, including cephalosporins and ampicillin, were detected only at modest or trace concentrations ranging from 0.03 to 4.18 ng/L. This trace-level occurrence is consistent with the chemical instability of the β-lactam ring, which is highly susceptible to rapid chemical and enzymatic hydrolysis in aquatic environments (Xiao et al., 2023).

### 3.2 Identification of isolated bacterial strains

The taxonomic identities of all 14 isolates were confirmed via 16S rRNA gene sequencing. These isolates were assigned to 11 different genera, indicating a relatively high level of cultivable microbial diversity in the water samples. The isolation results revealed specific associations between bacterial strains and sampling sites (Table 1). Specifically, *Bacillus subtilis* strain B11 was isolated from sample W1. *Acinetobacter johnsonii* strain B10 and *Klebsiella pneumoniae* strain B8 were both found in sample W2. *Pseudomonas putida* strain B3 was isolated from sample W3. *Klebsiella oxytoca* strain B6 was obtained from sample W4, while *Microbacterium* spp. strain B4 was isolated from sample W5. Additionally, *Acinetobacter oryzae* strain B9 was recovered from sample W7.

**Table 1:** Bacterial isolates from the respective source and identification.

Strain number	Sequencing results	Sequence derived strains	Similarity (%)	Source water sample
B3	*Pseudomonas putida*	*Pseudomonas knackmussii* (B13)	99.85	W3
B4	*Microbacterium spp*.	*Exiguobacterium undae* (DSM 14,481)	99.93	W5
B6	*Klebsiella oxytoca*	*Klebsiella oxytoca* (JCM 1665)	99.63	W4
B8	*Klebsiella pneumoniae*	*Klebsiella pasteurii* (SPARK836C1)	100.00	W2
B9	*Acinetobacter oryzae*	*Acinetobacter oryzae* (B23)	99.57	W7
B10	*Acinetobacter johnsonii*	*Acinetobacter johnsonii* (ATCC 17,909)	99.86	W2
B11	*Bacillus subtilis*	*Bacillus subtilis* (DMS 10)	100.00	W1
B12	*Foetidibacter luteolus*	*Foetidibacter luteolus* (WP_153796898.1)	98.80	W3
B13	*Polynucleobacter sp. AM-26B4*	*Polynucleobacter sp. AM-26B4* (WP_216249529.1)	99.10	W2
B14	*Limnohabitans sp. Bal53*	*Limnohabitans sp. Bal53* (WP_108362681.1)	100.00	W6
B15	*Paracoccus denitrificans*	*Paracoccus denitrificans* (IP29923)	99.10	W4
B16	*Thermus thermophilus*	*Thermus thermophilus* (JQ56218)	98.40	W1
B17	*Bacillus cereus*	*Bacillus cereus strain* (ATCC 14,579)	100.00	W2
B18	*Escherichia coli*	*Escherichia coli* (JP24183)	99.96	W1

At the genus level, *Bacillus, Klebsiella*, and *Acinetobacter* were the most frequently detected genera. Several species isolated in this study, including *Pseudomonas putida, Polynucleobacter* spp., and *Limnohabitans* spp., are commonly regarded as typical background commensals in freshwater ecosystems. Although not typically considered direct human pathogens, such environmental organisms play a critical role in the aquatic resistome, as they can serve as significant reservoirs for antibiotic resistance genes under chemical selective pressure (Yuan et al., 2019).

In contrast, species such as *Klebsiella pneumoniae, Klebsiella oxytoca*, and *Acinetobacter johnsonii* are well-documented opportunistic pathogens. Although primarily an environmental bacterium, *Bacillus cereus* is also a known foodborne and opportunistic pathogen. The detection of these opportunistic pathogens—particularly *Klebsiella pneumoniae*, which is frequently implicated in severe hospital- and community-acquired infections—highlights the potential biological risk in these water sources. When such pathogens coexist with anthropogenic antibiotic contamination, drinking water sources can become potential pathways for human exposure to resistant bacteria or resistance determinants.

### 3.3 Antimicrobial susceptibility of isolated strains

**Figure 2:**
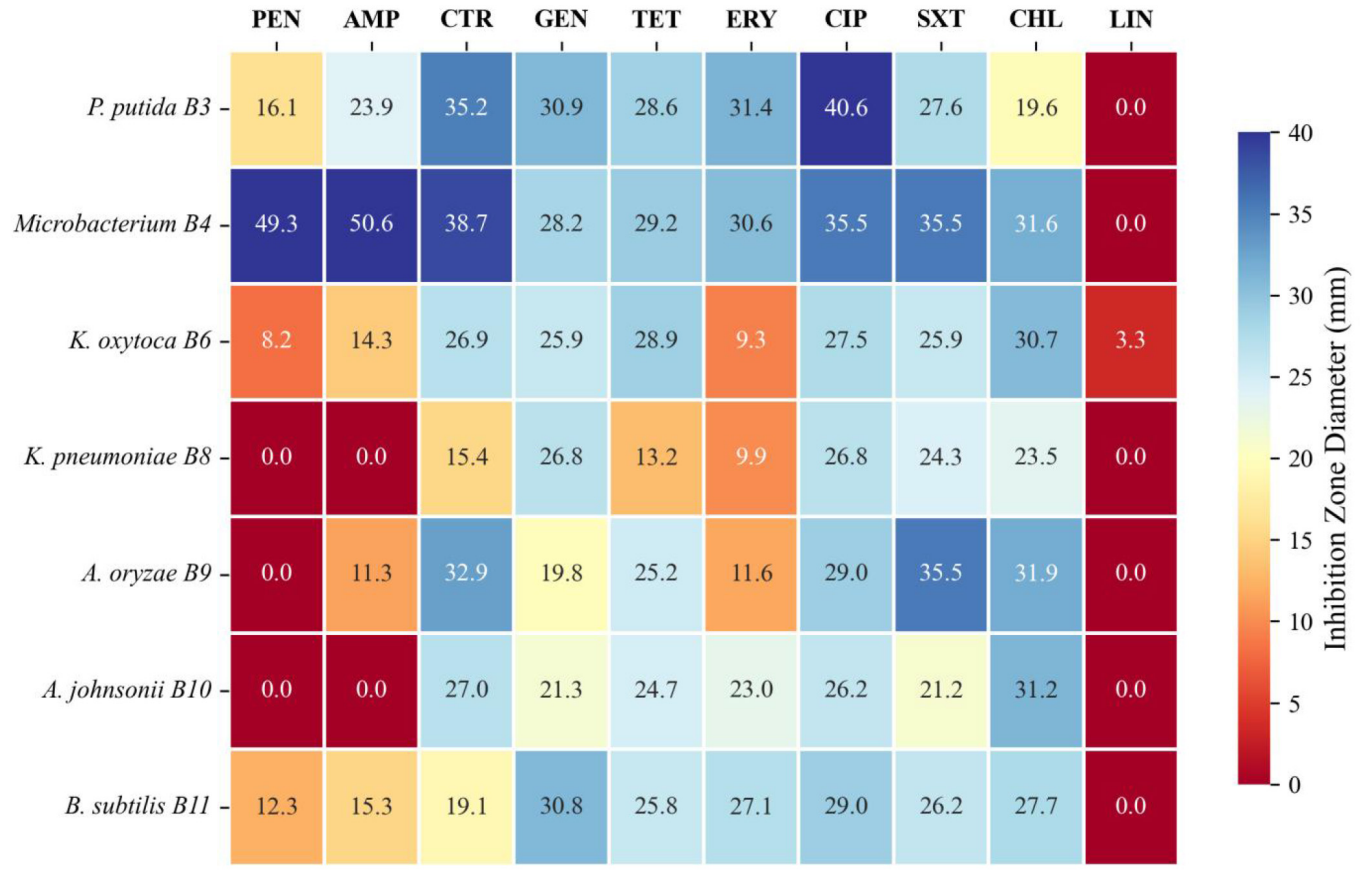
Antimicrobial susceptibility profile of seven representative bacterial isolates recovered from the water sources. The heatmap displays the diameter of inhibition zones (mm) for ten tested antibiotics. The color gradient from red to blue represents the transition from resistance (small or no inhibition zone) to susceptibility (large inhibition zone). *Klebsiella pneumoniae* strain B8 (highlighted in the fourth row) exhibits a distinct MDR pattern, contrasting with the susceptible environmental background isolates such as *Microbacterium* spp. B4.

Antimicrobial susceptibility of the seven representative bacterial isolates was evaluated using the disk diffusion assay (Figure 2). Notably, *Klebsiella pneumoniae* strain B8, which was recovered from the highly contaminated site W2, exhibited resistance to multiple tested antibiotics. This isolate showed complete resistance to penicillin and ampicillin (inhibition zone = 0.0 mm) and significantly reduced susceptibility to tetracycline (13.2 mm) and erythromycin (9.9 mm). The high-level resistance observed in *Klebsiella pneumoniae* isolated from an antibiotic contamination hotspot is consistent with localized environmental selective pressure (Raed, 2017).

In contrast, other isolates generally demonstrated higher degrees of susceptibility. *Pseudomonas putida* B3 and *Microbacterium* spp. B4 displayed moderate inhibition zones against ceftriaxone and tetracycline. *Acinetobacter oryzae* B9 and *Acinetobacter johnsonii* B10 were highly susceptible to gentamicin and ciprofloxacin, exhibiting inhibition zone diameters exceeding 25 mm. These differing profiles indicate that while specific opportunistic pathogens like *Klebsiella pneumoniae* maintain clinically relevant resistance, the broader environmental microbiome in these water sources remains largely susceptible to several antibiotic classes, likely due to the fitness costs associated with maintaining resistance in the absence of lethal concentrations (Karkman et al., 2018).

### 3.4 Microbial community structure and diversity in water samples

#### 3.4.1 Taxonomic composition

**Figure 3:**
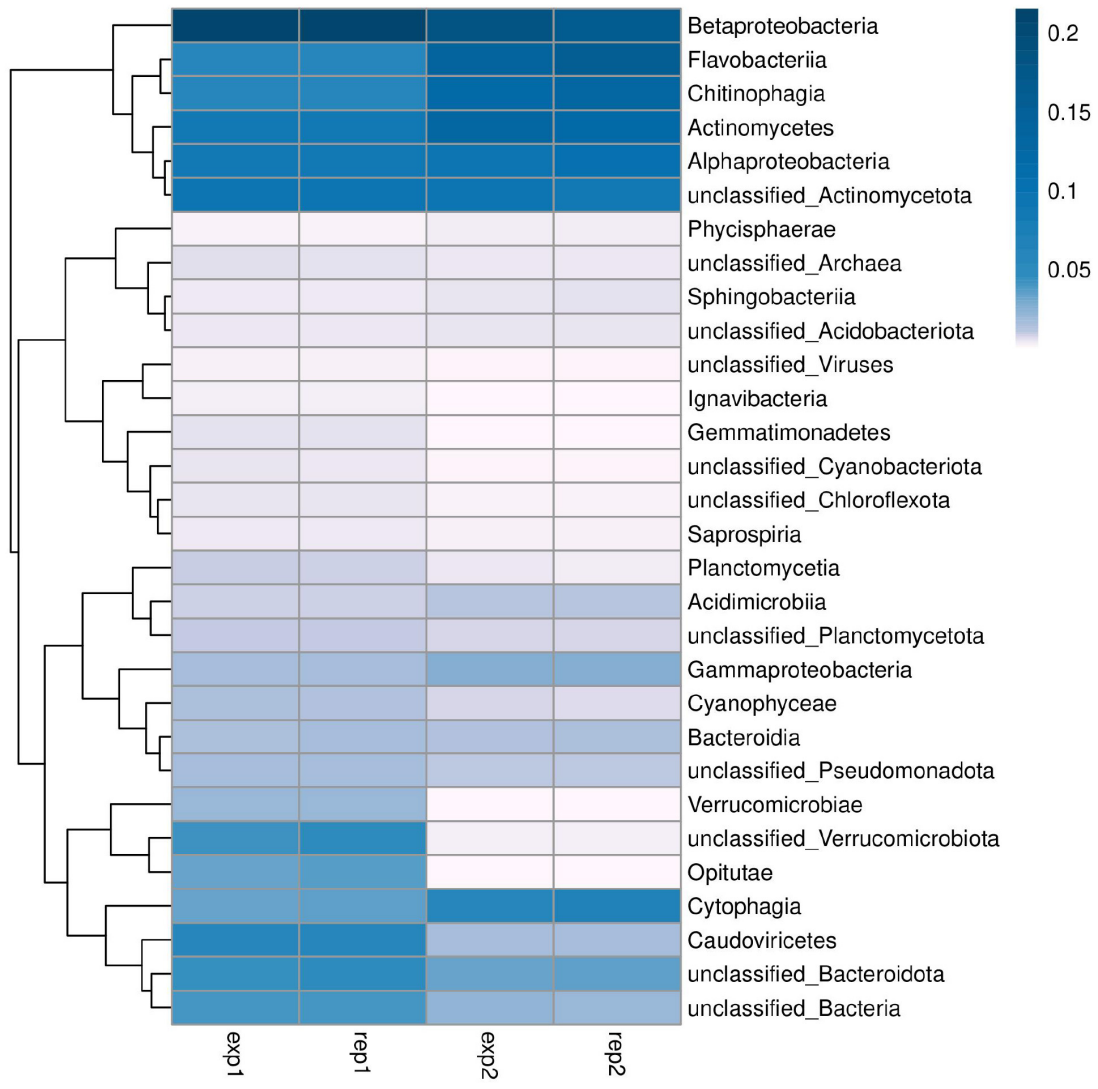
Heatmap analysis of the microbial community composition at the genus level. The plot displays the relative abundance of the top 30 genera identified in samples W1 (exp1, rep1) and W2 (exp2, rep2). The color gradient reflects the proportion of each taxon, with darker blue indicating higher prevalence.

Metagenomic sequencing of the surface water samples revealed a diverse microbial community, which was predominantly composed of the phyla Proteobacteria, Actinobacteriota, and Bacteroidota. Together, these three phyla accounted for more than 90% of the total relative abundance across the investigated sites. At the genus level, the taxonomic profile was characterized by the dominance of *Limnohabitans* (9.35% in W1 and 8.88% in W2) and various unclassified Actinobacteriota (9.22% in W1 and 8.94% in W2), as illustrated in the relative abundance heatmap (Figure 3).

The methodological divergence between metagenomic quantification and culture-based recovery of the genus *Klebsiella*. While sequences assigned to *Klebsiella* were detectable within the metagenomic datasets of both W1 and W2, they represented a minor component of the “rare biosphere,” with a relative abundance consistently below 0.2%. Despite this low genomic representation, the viable and multidrug-resistant *Klebsiella pneumoniae* strain B8 was successfully isolated from the W2 sample matrix. This discrepancy suggests that rare opportunistic pathogens may be overlooked in abundance-based analyses while remaining viable in the environment within the aquatic environment. Such findings support the value of integrating culture-based methods with metagenomic analysis, emphasizing that culture-dependent validation is essential for identifying high-risk pathogens that might be overlooked by DNA sequencing alone (Bai et al., 2019).

#### 3.4.2 Alpha and beta diversity

The complexity of the microbial communities was further evaluated through diversity indices. As summarized in Table 2, both samples exhibited substantial species richness and evenness. The Chao1 index exceeded 4,500 and the Shannon index remained above 6.0 for all replicates, while Good's coverage values approaching 1.0 indicated that the sequencing depth was sufficient to capture the vast majority of the taxonomic diversity present in the source water. No statistically significant differences in alpha diversity were observed between sites W1 and W2, suggesting a relatively stable baseline of species richness across these karst hydrologic points.

**Table 2:** Statistical summary of alpha diversity indices for metagenomic samples.

Sample	Ace	Chao1	Shannon	Simpson	Goods_coverage
exp1	4,614.628	4,611.316	6.323	0.968	1
rep1	4,543.075	4,535.265	6.299	0.969	1
exp2	4,500.144	4,480.474	6.093	0.965	1
rep2	4,390.871	4,397.307	6.06	0.965	1

Indices including ACE, Chao1, Shannon, and Simpson were calculated to assess the richness and diversity of the microbial communities in W1 and W2.

**Figure 4:**
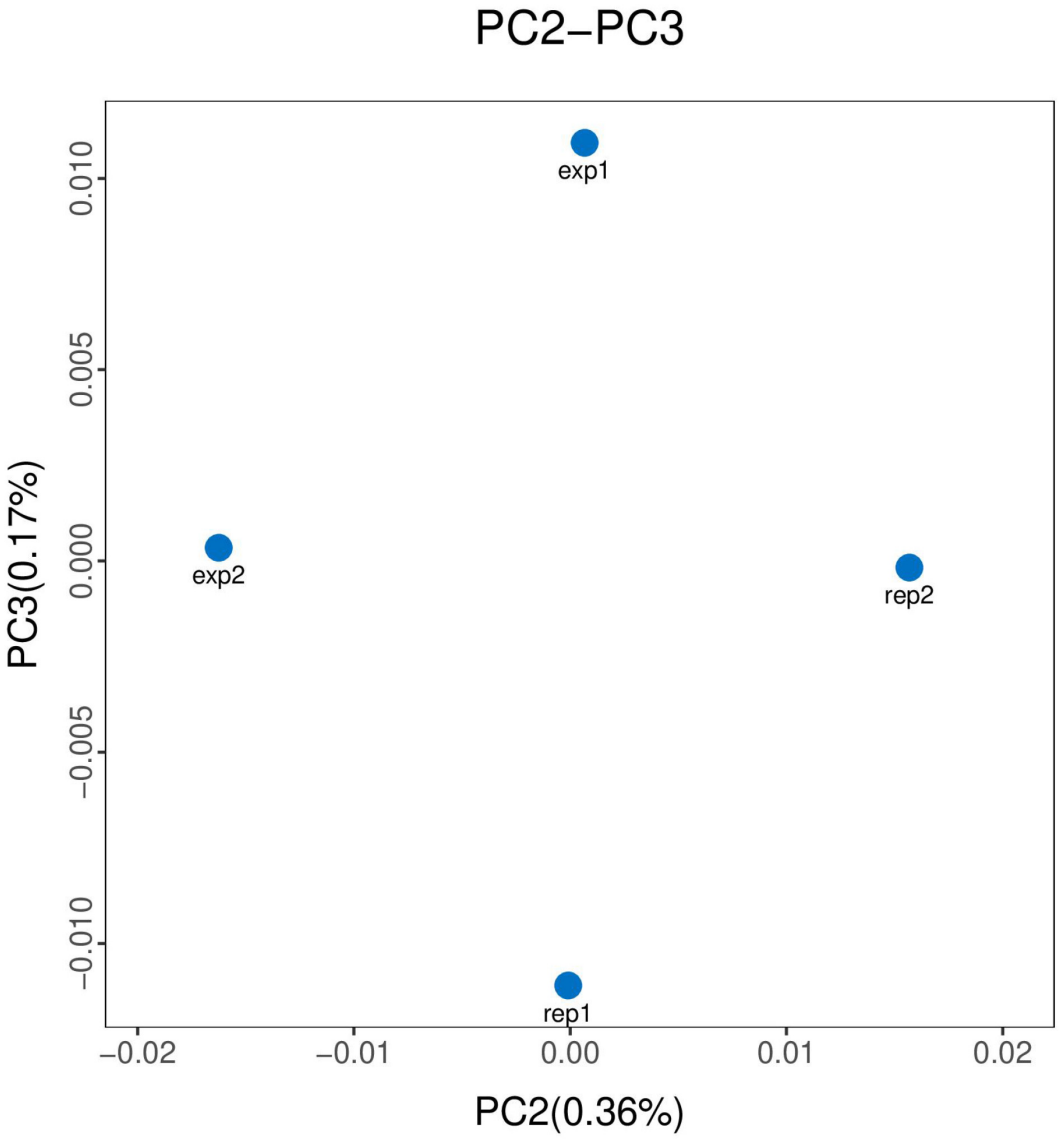
Principal Coordinates Analysis (PCoA) of microbial communities based on Bray–Curtis dissimilarity. The plot visualizes the structural separation between samples W1 and W2. The percentages on the axes represent the proportion of variance explained by the first two principal coordinates.

However, the community structure exhibited significant spatial differentiation when analyzed through beta diversity metrics. Principal coordinates analysis (PCoA) based on Bray–Curtis dissimilarity (Figure 4) clearly separated the communities of W1 and W2 along the primary axis. While technical replicates clustered tightly together—demonstrating high experimental reproducibility—the distinct separation between sampling sites suggests that localized environmental factors, likely including the specific antibiotic contamination profiles identified in section 2.1, play a significant role in shaping the specific composition of the water source microbiome.

### 3.5 Resistome characteristics of water samples

#### 3.5.1 Functional profiling and antibiotic resistance genes

**Figure 5:**
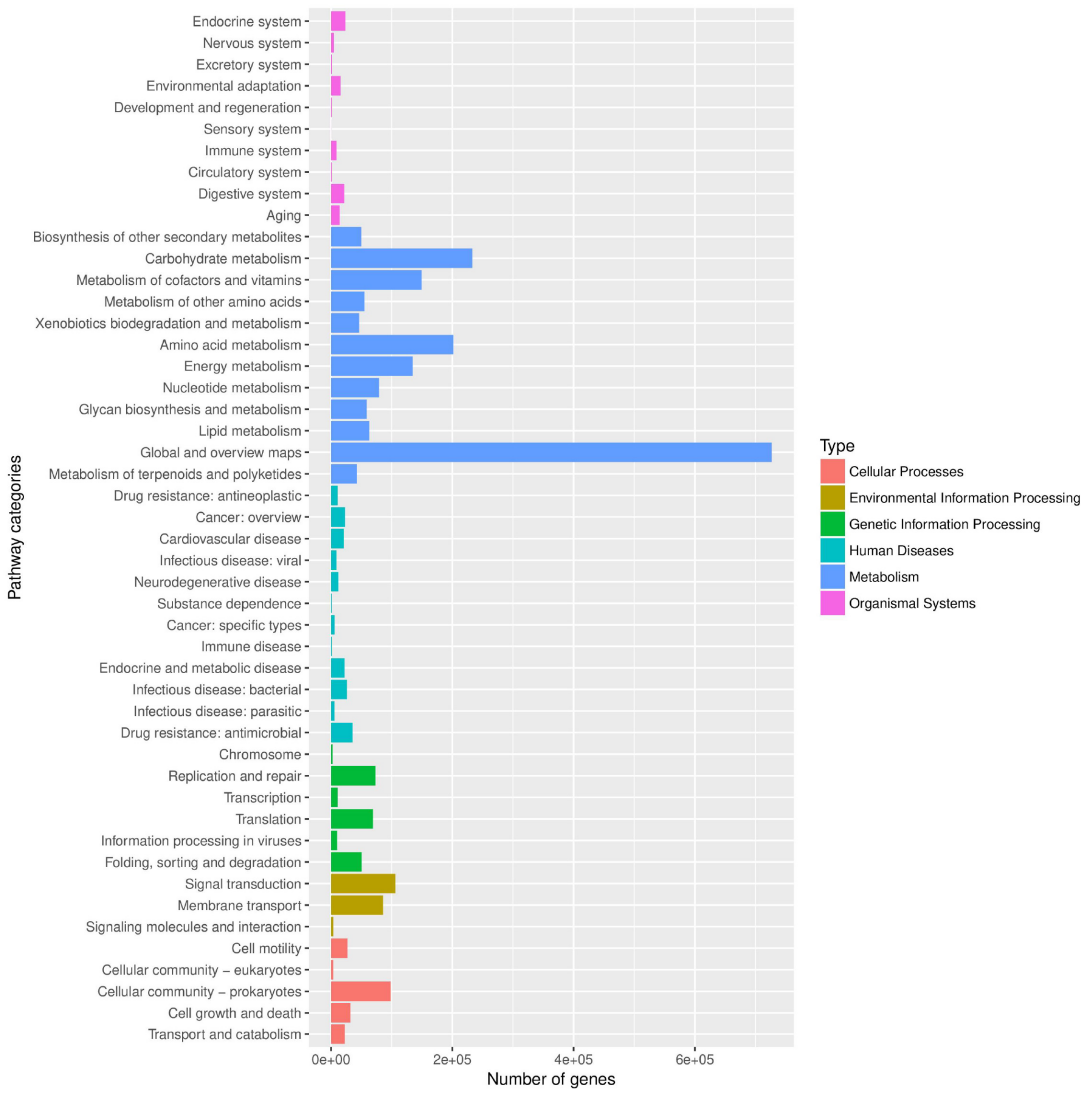
KEGG pathway classification. The y-axis represents the secondary classification of biological pathways; the x-axis represents the number of genes. Different colors indicate the primary functional categories of biological pathways.

**Figure 6:**
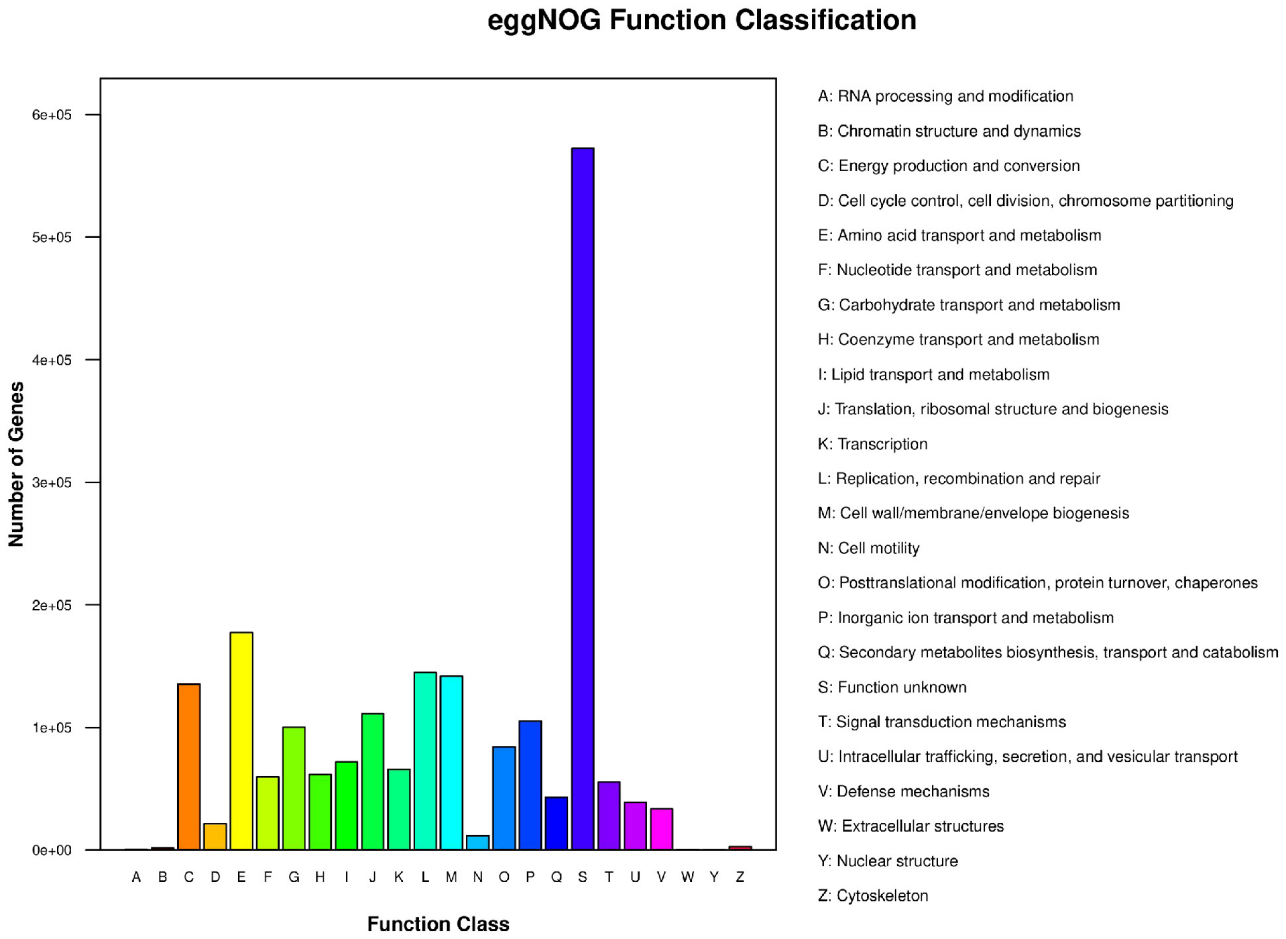
Functional classification of predicted genes based on the eggNOG database. The x-axis represents the functional categories of eggNOG, and the y-axis represents the number of genes annotated to each category. The specific definition of each category code is provided in the legend on the right.

To assess the metabolic potential of the microbial communities, gene sequences were annotated against the KEGG and eggNOG databases. As shown in Figure 5, the functional landscape was dominated by metabolism-related pathways, particularly “Global and overview maps” and “Carbohydrate metabolism,” indicating a metabolically active community capable of responding to environmental stimuli. Complementing this, the eggNOG functional classification (Figure 6) revealed a high prevalence of genes associated with “Amino acid transport and metabolism” (Category E) and “Energy production and conversion” (Category C). This robust metabolic profile suggests that the resident microbiota are well-adapted for persistence in the aquatic environment (Qingxiang et al., 2016).

Specific profiling against the Comprehensive Antibiotic Resistance Database (CARD) revealed a diverse array of ARGs. Consistent with the antibiotic residues detected, such as sulfachloropyridazine and chlortetracycline, genes conferring resistance to sulfonamides (such as *sul1* and *folP*) and tetracyclines (such as *tet(Q)*) were frequently identified. Furthermore, the analysis identified genes encoding resistance to glycopeptides (*vanP*) and isoniazid (*katG*), indicating a broader resistome than that suggested by chemical analysis alone (Elizabeth et al., 2016).

#### 3.5.2 Functional clustering and potential dissemination risk

**Figure 7:**
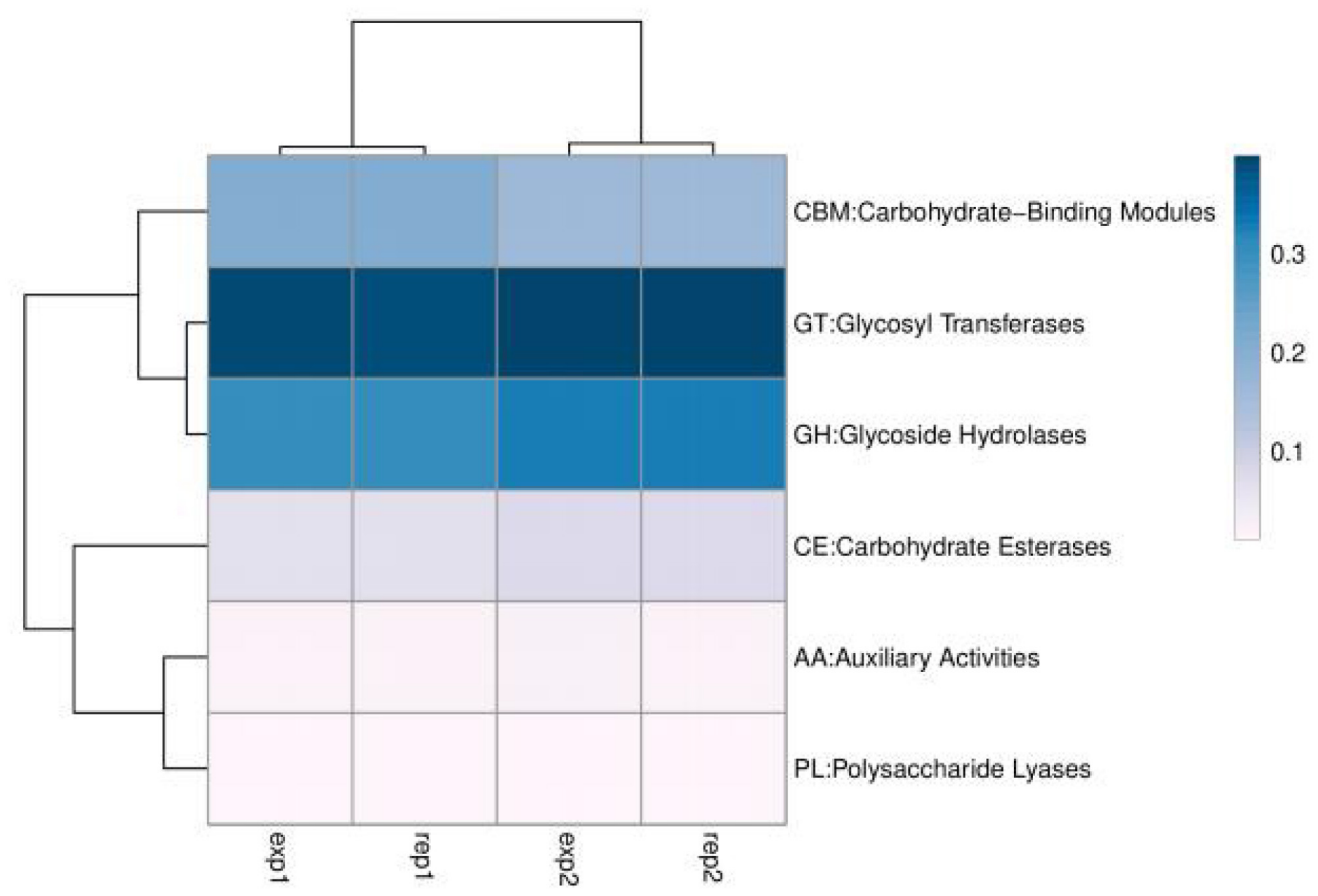
Heatmap of functional abundance clustering. The heatmap illustrates the co-occurrence patterns of identified functional gene categories across samples. The clustering dendrogram **(left)** groups functions with similar abundance profiles, highlighting the potential association between resistance determinants and mobile genetic elements.

To evaluate the potential dissemination risk of ARGs in the aquatic environment, functional clustering analysis was performed based on annotation results. The functional abundance heatmap (Figure 7) showed that annotated ARGs clustered closely with specific functional categories, including DNA transposition, recombination and repair, and prokaryotic defense mechanisms. In particular, ARGs mediating resistance to tetracyclines and sulfonamides clustered within the same or adjacent branches as functional groups containing key mobile genetic element (MGE) components, such as transposases and integrases (Sarah et al., 2016).

This functional co-clustering analysis suggests a potential association between ARG abundance and MGE-related functional signatures. Specifically, the spatial proximity of *sul1* and *tet* genes to transposase signatures in the functional network suggests that these resistance determinants may be associated with mobile genetic elements. Such genetic arrangements possess a high potential for horizontal dissemination among the aquatic microbial community (Knupp et al., 2015), potentially transferring multidrug-resistant traits to opportunistic pathogens like *Klebsiella pneumoniae*.

### 3.6 tetA gene expression in *Klebsiella pneumoniae* strain B8

To mechanistically corroborate the multidrug-resistant phenotype of *Klebsiella pneumoniae* strain B8, particularly its reduced susceptibility to tetracycline, the transcriptional expression of the *tetA* efflux pump gene was quantified via RT-qPCR. The specificity of the amplification was verified through melt curve analysis, which confirmed the absence of non-specific products.

**Figure 8:**
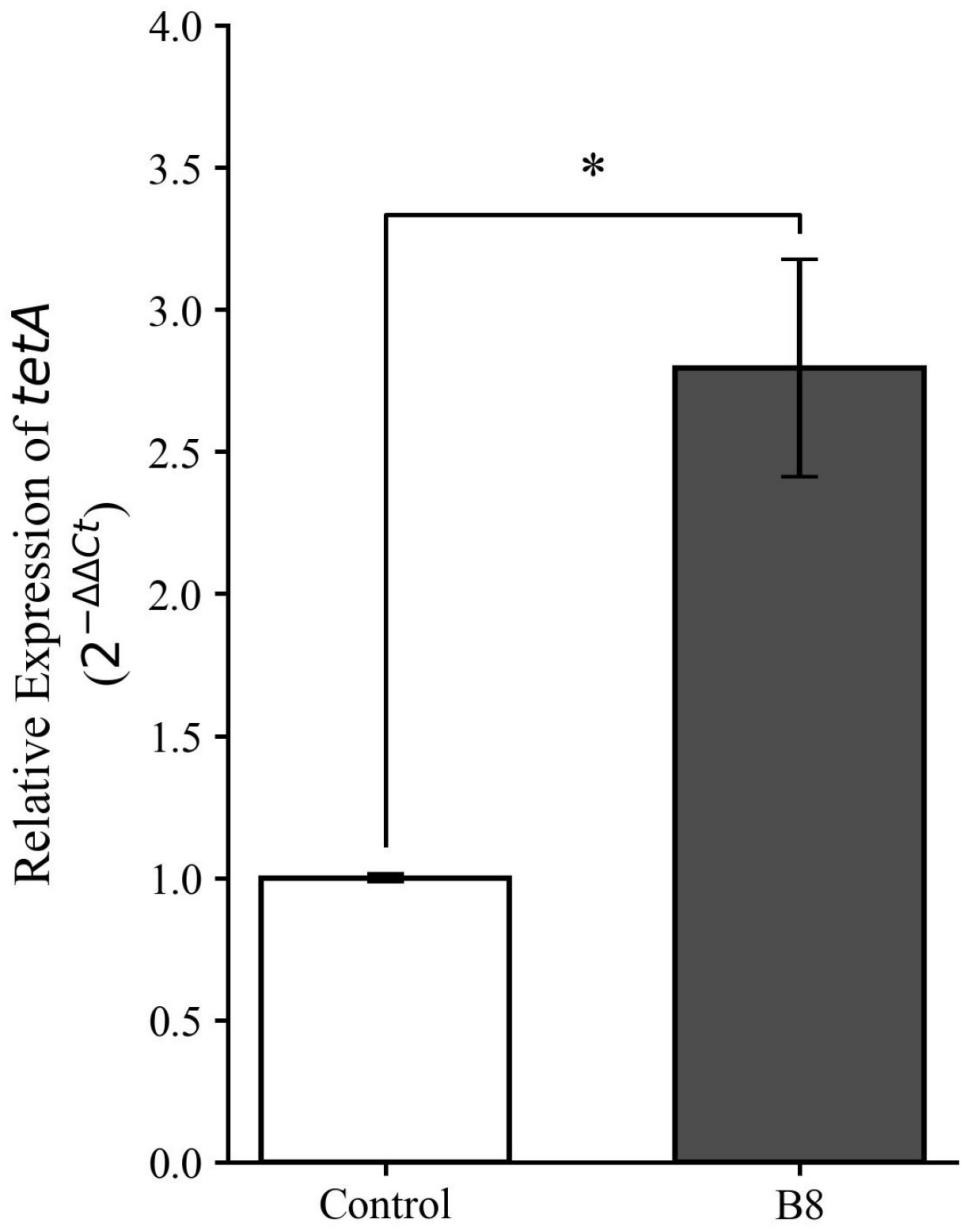
Relative expression levels of the antibiotic resistance gene *tetA* in the environmental isolate *Klebsiella pneumoniae* strain B8. The y-axis represents the relative fold change in gene expression calculated using the comparative 2^−ΔΔCt^ method, normalized to the 16S rRNA endogenous reference gene. Data are presented as the mean of three independent biological replicates, with error bars indicating the standard deviation. The asterisk denotes a statistically significant difference between the isolate and the reference control (*p* < 0.05).

The quantitative results (Figure 8) provide molecular evidence of active resistance expression. *Klebsiella pneumoniaestrain* B8 exhibited a significant 2.78-fold higher relative expression of the *tetA* gene (*p* < 0.05) compared to the baseline reference control. This elevated transcriptional is consistent with the solate's recovery from the multi-contaminated source water of sample W2. This molecular validation suggests that *tetA* expression may contribute to the observed tetracycline resistance phenotype of the efflux pump, a common adaptive strategy for pathogens surviving in complex chemical environments (Karkman et al., 2018).

### 3.7 Ecological concordance among chemical pressure, resistome, and phenotype

**Figure 9:**
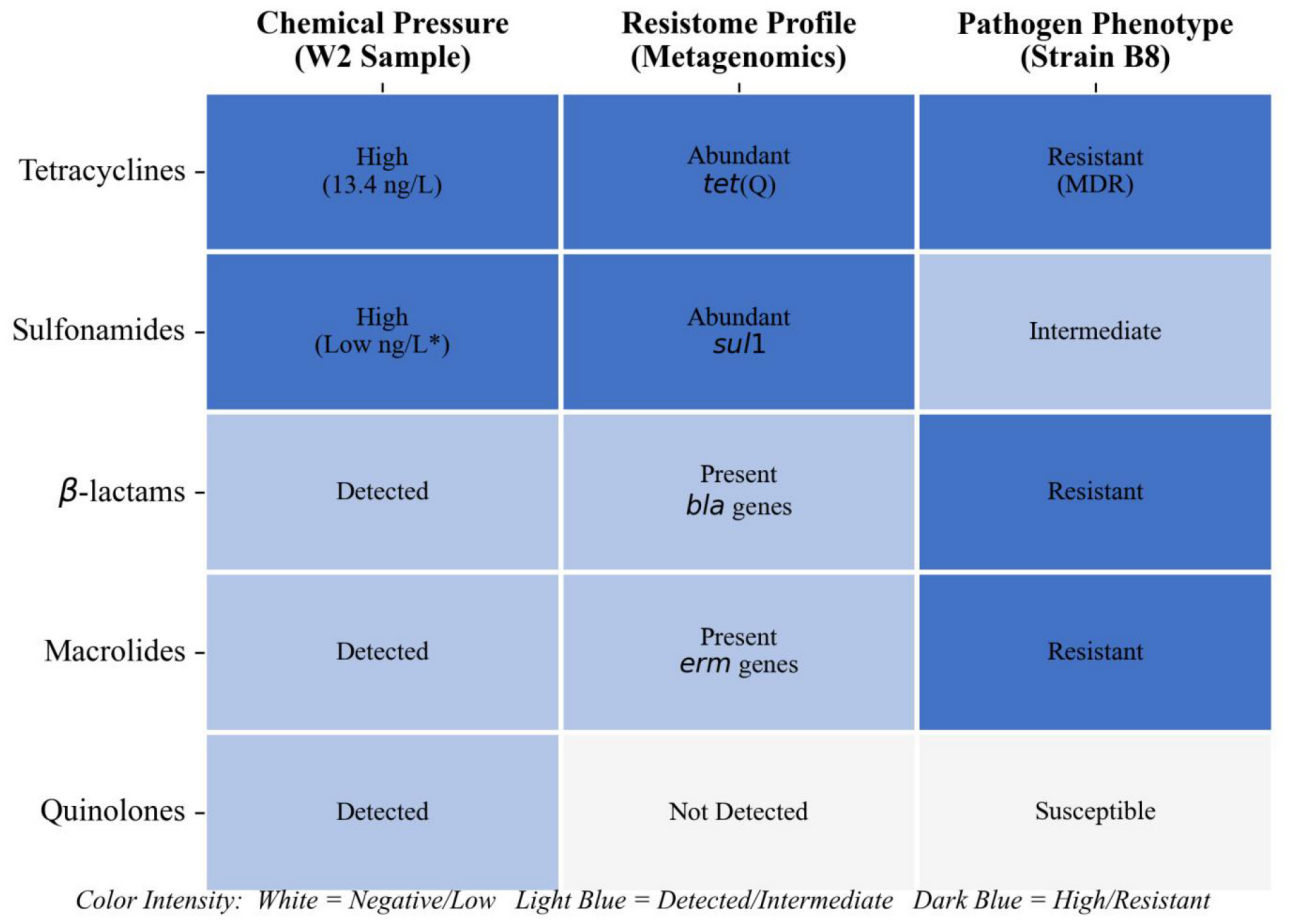
Ecological concordance matrix illustrating the multi-level associations in the drinking water source (Sample W2). The matrix correlates three layers of evidence: (1) Chemical Pressure: antibiotic residues detected via LC-MS/MS; (2) Resistome Profile: presence and abundance of ARGs identified via metagenomic sequencing; and (3) Pathogen Phenotype: antimicrobial susceptibility profile of *Klebsiella pneumoniae* strain B8. The alignment highlights the complete chain of evidence from environmental contamination to genotypic reservoir and specific pathogenic expression.

To synthesize the multi-tiered findings of this study, an ecological concordance matrix (Figure 9) was constructed to connect the chemical selective pressure, the metagenomic reservoir of resistance genes, and the specific phenotypic traits of the isolated pathogen (Amos et al., 2014).

An apparent ecological concordance was observed, particularly within the multi-contaminated Sample W2. Chemical profiling of this matrix detected a complex mixture of antibiotics, including tetracyclines (chlortetracycline, 13.35 ng/L), sulfonamides, macrolides, and β-lactams (such as penicillin G and cefoperazone; Huang et al., 2014). This specific chemical profiling was broadly consistent with the metagenomic landscape, where genes conferring resistance to these exact classes (such as *tet(Q), sul1, erm*, and *bla variants*) were abundantly identified (Su et al., 2014).

Crucially, the environmental resistome was consistent with the resistance phenotype observed in *Klebsiella pneumoniae* strain B8, which was isolated from this exact site. Strain B8 exhibited a robust multidrug-resistant profile, characterized by complete resistance to penicillin and ampicillin, and reduced susceptibility to tetracycline and erythromycin, matching the dominant contaminants. Conversely, although quinolones were present in W2, strain B8 remained highly susceptible to ciprofloxacin. This difference suggests that resistance was not uniformly expressed across all tested antibiotic classes demonstrating that the observed multidrug resistance is a targeted adaptive response to localized selective pressures rather than a generalized tolerance mechanism. Collectively, Figure 9 visualizes how multi-class chemical contaminants can maintain a corresponding resistome, which subsequently governs the physiology of opportunistic pathogens in drinking water sources (Karkman et al., 2018).

## 4 Discussion

### 4.1 Concordance between chemical selection and the environmental resistome

This case study provides a focused analysis of the linkages between anthropogenic pollutants and microbial adaptation within a specific karst drinking water system. Unlike broad surveillance studies, our integrated approach highlights a specific correlation between chemical selection pressures and the genomic composition of the microbiome. In the specific case of Sample W2, the detection of chlortetracycline (13.35 ng/L) and sulfachloropyridazine was mirrored by the metagenomic abundance of corresponding resistance determinants, specifically *tet(Q)* and *sul1*. This alignment, visualized in the ecological concordance matrix (Figure 9), suggests that even trace-level antibiotic residues (ng/L range) may act as selective forces contributing to the maintenance of specific resistance genotypes in the local resistome. While the microbial community structure was visually dominated by typical freshwater commensals such as Limnohabitans, the “rare biosphere” harbored potential biological risks. The functional profiling (KEGG and eggNOG) further indicated that this community possesses a robust metabolic potential, which may create a cellular environment conducive to the active replication and dissemination of these resistance elements, rather than them merely existing as dormant extracellular DNA (Jing et al., 2013).

### 4.2 Klebsiella pneumoniae as a sentinel of pathogenic risk

The recovery of the multidrug-resistant *Klebsiella pneumoniae* strain B8 provides biological evidence in this case study, highlighting a link between the environmental resistome and potential health risks (Yan et al., 2013). While metagenomics identified the “potential” risk (presence of *Klebsiella*-specific reads and virulence factors like *gspE* and *GroEL*), the successful isolation of viable *Klebsiella pneumoniae* indicates that these opportunistic pathogens are present and physiologically competent to survive environmental stressors. The co-occurrence of virulence determinants and ARGs within the same environmental matrix supports the hypothesis that drinking water sources may function as “co-selection arenas.” In this specific ecological niche, *Klebsiella pneumoniae* could act as a reservoir where resistance traits and pathogenicity factors (e.g., Type II secretion systems) converge, potentially compounding the public health risk if these strains are transmitted to human hosts through the water supply chain (Yan et al., 2013).

### 4.3 Mechanistic validation of the genotype-phenotype link

A limitation in many environmental studies is the reliance on genomic prediction without phenotypic verification. This case study addresses that limitation by providing mechanistic insights into resistance expression. The significant 2.78-fold upregulation of the *tetA* efflux pump gene in *Klebsiella pneumoniae* strain B8 (Figure 8) provides a potential molecular basis for its survival in the tetracycline-impacted W2 environment. These molecular data indicate that the “environmental resistome” involves dynamic, expressible traits in viable opportunistic pathogens (Magdalena et al., 2012). Furthermore, the functional clustering analysis (Figure 7) revealed that these resistance genes are spatially associated with mobile genetic elements (such as transposases and integrases). This proximity suggests a potential for horizontal gene transfer, indicating that *Klebsiella pneumoniae* might participate in the dissemination of these *tet* and *sul* genes to other microbiota. Consequently, this case study suggests that relying solely on chemical monitoring or metagenomics may be insufficient. Integrated surveillance—which triangulates chemical pressure, genomic reservoirs, and pathogen viability—is essential for comprehensively assessing the biological safety of drinking water sources (Bruno, 2010).

### 4.4 Public health implications and policy recommendations

The isolation of multidrug-resistant *Klebsiella pneumoniae* from a karst drinking water source indicates a potential exposure pathway for the local population. However, the presence of ARGs and resistant opportunistic pathogens in surface waters does not directly predict clinical outbreaks; rather, it highlights a silent dissemination of resistance traits that could complicate long-term public health management. Given the high permeability of karst aquifers, conventional water quality metrics may not fully capture the biological risks associated with the environmental resistome (Surette and Wright, 2017).

Based on these specific correlations, we propose several restrained policy recommendations for karst drinking water management. First, regional water quality monitoring frameworks should be gradually expanded to incorporate a “One Health” approach, combining routine microbiological tests with the surveillance of representative antibiotic residues and ARG markers (such as *tetA* and *sul1*). Second, as sulfonamides and tetracyclines were frequently detected, source-control policies should be strengthened to manage agricultural runoff and untreated livestock wastewater in upstream catchment areas. Finally, upgrading rural decentralized wastewater treatment systems could help mitigate the continuous influx of these micropollutants, ultimately reducing the selective pressure on the aquatic microbiome.

## 5 Limitations

Despite providing critical insights into the karst aquatic resistome, this study has several limitations. First, metagenomic sequencing was strategically prioritized for the highly contaminated hotspots (samples W1 and W2) identified during the initial chemical screening. While this targeted approach effectively characterized the most critical environmental risks, it limited the high-resolution profiling of spatial and temporal resistance dynamics across the broader background monitoring sites.n addition, although seven monitoring sites were initially surveyed, metagenomic sequencing and mechanistic validation were ultimately concentrated on the two hotspot samples and a single representative isolate, which limited the overall sample size and may affect the generalizability of the findings.

Second, the reliance on traditional culture-dependent methods for phenotypic validation inherently introduces cultivation bias. Consequently, the obtained isolates, including the multidrug-resistant *Klebsiella pneumoniae*, represent only a fraction of the environmental microbiome, potentially excluding viable but non-culturable organisms that may also harbor and disseminate ARGs.

Finally, although the functional co-clustering of ARGs and mobile genetic elements strongly indicates a high potential for horizontal gene transfer, this study did not perform *in vitro* conjugation assays to experimentally confirm the physical mobility of these resistance determinants. Furthermore, the mechanistic validation via RT-qPCR was explicitly focused on the *tetA* efflux pump in a single representative isolate. Future investigations should incorporate broad-spectrum metatranscriptomics and experimental conjugation models to fully elucidate the active transmission networks of the resistome in karst hydrologic systems.

## 6 Conclusion

This integrated case study provides an evidence chain linking environmental chemical pressures to the physiological adaptation of opportunistic pathogens in a karst drinking water source. Our results demonstrate a multi-level concordance: the chemical persistence of sulfonamides and tetracyclines (such as chlortetracycline) was mirrored by the metagenomic enrichment of *sul1* and *tet* genes, which ultimately manifested as a viable, multidrug-resistant phenotype in the isolated *Klebsiella pneumoniae* strain B8. The molecular validation of *tetA* overexpression further confirmed that these resistance mechanisms are functionally active (Riesenfeld et al., 2004). These findings indicate that drinking water sources can act as silent reservoirs where the environmental resistome and clinical pathogenome intersect, potentially facilitated by mobile genetic elements. Consequently, this study highlights the necessity for an integrated water quality monitoring framework that moves beyond simple chemical detection to include resistome profiling and the culture-based verification of high-risk pathogens like *Klebsiella pneumoniae*. Such a comprehensive approach is crucial for evaluating and mitigating the public health risks posed by waterborne antibiotic resistance.

## Data Availability

The raw data supporting the conclusions of this study are not publicly available due to institutional and confidentiality restrictions. Requests to access the data should be directed to the corresponding author.
